# Blood glucose regulation in type 1 diabetic patients: an adaptive parametric compensation control‐based approach

**DOI:** 10.1049/iet-syb.2017.0093

**Published:** 2018-10-01

**Authors:** Anirudh Nath, Dipankar Deb, Rajeeb Dey, Sipon Das

**Affiliations:** ^1^ Electrical Engineering Department National Institute of Technology Silchar 788010 Assam India; ^2^ Electrical Engineering Department Institute of Infrastructure Technology Research and Management Ahmedabad 380026 Gujarat India

**Keywords:** patient monitoring, adaptive control, diseases, Lyapunov methods, closed loop systems, medical control systems, patient treatment, medical computing, sugar, uncertain systems, blood, nonlinear control systems, physiological models, blood glucose regulation, type 1 diabetic patients, adaptive parametric compensation control‐based approach, direct adaptive control strategy, nonlinear model, type 1 diabetes mellitus patients, uncertain parameters, appropriate design, adaptation laws, closed‐loop response, plasma glucose concentration, external insulin infusion rate, model parameters, adaptive control scheme, parametric uncertainty, inter‐patient variability

## Abstract

Here, a direct adaptive control strategy with parametric compensation is adopted for an uncertain non‐linear model representing blood glucose regulation in type 1 diabetes mellitus patients. The uncertain parameters of the model are updated by appropriate design of adaptation laws using the Lyapunov method. The closed‐loop response of the plasma glucose concentration as well as external insulin infusion rate is analysed for a wide range of variation of the model parameters through extensive simulation studies. The result indicates that the proposed adaptive control scheme avoids severe hypoglycaemia and gives satisfactory performance under parametric uncertainty highlighting its ability to address the issue of inter‐patient variability.

## 1 Introduction

Blood glucose (BG) concentration in a healthy person is regulated within a safe range of 70–180 mg/dl due to insulin secretion by the pancreas. When there is an autoimmune destruction of pancreatic β‐cells, negligible or no such secretion takes place in human body, leading to a disease called type 1 diabetes mellitus (T1DM). Patients suffering from T1DM have impaired glucose–insulin regulation mechanism, leading to prolonged hyperglycaemia (glucose level >180 mg/dl) [1]. To avoid this situation, the patients rely on multiple daily insulin injections in an attempt to restore normal glucose concentration level. As this manual (or open‐loop), insulin therapy is based on irregular glucose measurement, thus at certain instances, due to improper insulin dosages, glucose concentration can drastically fall <50 mg/dl, leading to a situation called hypoglycaemia. The hypoglycaemic situation in T1DM patients can cause hypoglycaemic coma and death, whereas hyperglycaemia can lead to long‐term complications like cardiac arrests, leg amputations, renal failure, and diabetic retinopathy [2]. To circumvent these situations, a closed‐loop control strategy is required for continuous glucose measurement, which is realised through an artificial pancreas system (APS) [3] to mimic glucose–insulin homoeostasis artificially.

The main research for APS involves the design of an efficient closed‐loop control strategy based on the mathematical model of the T1DM patient. The patient models are classified as intravenous and subcutaneous [4]. In the present work, widely used Bergman's minimal model [5] is considered as an intravenous patient model. This model is also referred in the literature as intravenous glucose tolerance test (IVGTT) model. In reality, model parameters are uncertain and vary from patient to patient (called as inter‐patient variability) as well as within the same patient (called as intra‐patient variability) owing to the variation of physiological parameters like insulin sensitivity, exercise, stress, infection, and food intake [6]. Significant challenges arise in the design of closed‐loop control for BG regulation when model parameters are uncertain. In the present work, the problem is solved with a new adaptive control strategy.

Some of the adaptive control strategies [7–9] applied or developed for this problem are summarised here in brief. Standard adaptive control strategies like minimum variance control [10, 11] and self‐tuning regulator [12–14] in conjunction with parameter estimation via Kalman filtering and recursive least square methods were incorporated for this problem. Adaptive linear quadratic Gaussian controllers have been proposed in [15, 16] for Dalla Man's subcutaneous model [17] and Hovork's model [18]. Both IVGTT and subcutaneous‐based model reference adaptive control (MRAC) techniques exist [19–22] where MRAC is implemented in conjunction with (i) modified smith‐predictor structure [20], and (ii) adaptive disturbance rejection [21]. Apart from these algorithms, an adaptive controller with online parameter adaptation [23] and a non‐linear adaptive control method based on exact feedback linearisation [24] was proposed. In the recent past, a wide range of adaptive model predictive controllers (MPC) have been proposed for BG regulation [18, 25–27]. Careful study of the methodology and philosophy of these methods reveals inherent deficiencies in these control methods: (i) aggressive control leading to hypoglycaemic events [10, 11], (ii) use of linearised version of non‐linear physiological model leading to significant loss of information related to non‐linear system characteristics [12–16, 19–22] and (iii) in some cases, model parameters do not convey any physiological significance (such as information about insulin sensitivity) explicitly [10–14].

In the present work, a new adaptive state feedback control for a non‐linear IVGTT model is designed via Lyapunov stability theory. The proposed control technique has the following advantages: (i) unlike most of the adaptive control methods, the proposed approach is based on a non‐linear IVGTT minimal model that can express important physiological parameters (like insulin sensitivity) in terms of model parameters, (ii) no cost function is associated with the control law to give closed‐form solutions due to the use of Lyapunov theory, and (iii) unlike other MRAC schemes, here a non‐linear reference model is considered.

The modifications of the proposed control technique make it different from [28, 29] are

- The uncertainties are considered in the model parameters, whereas in [28, 29], uncertainties were in the actuator dynamics.
- The reference models were based on linear models, whereas a non‐linear model is used here.
- For the first time, adaptive parametric compensation is done for the model parameters unlike in [28, 29] for a non‐linear T1DM patient model. The control law has guaranteed stability where the model parameters are adapted online by adaptation laws.
- Unlike in [28, 29], here two sets of constant terms are introduced, $c_{i},\;i=1,\ldots ,4$, to impose constraints on the closed‐loop stability and $\gamma_{i}\;s$ to facilitate improvement in transient response. The tuning of $\gamma_{i}\;s$ are heuristically done from the expert knowledge of patients’ physiology.
- The proposed control technique avoids any occurrences of hypoglycaemic events in the presence of ±30% parametric uncertainty as well as multiple external meal disturbances for 200 virtual T1DM patients which are validated through simulation studies.

This paper is structured into five sections. Section 2 contains the problem formulation constituting of the system description and the control objectives. The adaptive controller is presented in Section 3. The simulation results and discussion constitute Section 4, and concluding remarks are provided in Section 5.

## 2 Problem formulation

In this section, a brief introduction on the physiological IVGTT model for the T1DM patient is presented in the first subsection. In the next subsection, the main objectives of the control algorithm are clearly stated.

### 2.1 Mathematical model of T1DM patients

A modified version of Bergman's minimal model as reported in [30, 31] that offers a good trade‐off between glucose–insulin response and model complexities has been taken into account. It has numerous applications in clinical trials [32] as well as intensive care unit medication systems [33]. The model is further modified by considering the meal disturbance dynamics as reported in [34], as the fourth state of the state‐space model in (1) as presented below:

(1) $$\begin{array}{rl} & {\dot{x}}_{1}=-p_{1}(x_{1}-G_{b})-x_{1}x_{2}+x_{4}\\ & {\dot{x}}_{2}=-p_{2}x_{2}+p_{3}(x_{3}-I_{b})\\ & {\dot{x}}_{3}=-p_{4}(x_{3}-I_{b})+u(t)\\ & {\dot{x}}_{4}=-p_{5}x_{4} \end{array}$$

where the state variables $x_{i},i=1,\ldots ,4$, represent the BG concentration (mg/dl), the remote insulin (min^−1^), plasma insulin concentration (mU/l), and the meal disturbance (mg/dl/min), respectively, given in (1), and $G_{b}$ and $I_{b}$ represent the basal value of plasma glucose concentration and plasma insulin concentration, respectively.

The first dynamical equation represents the plasma glucose compartment corresponding to the plasma glucose dynamics. The second differential equation accounts for the delayed action of insulin on the glucose dynamics in the body, and the third equation represents the plasma insulin kinetics where the control input (external insulin infusion) u(t) appears. The meal disturbance $x_{4}$ represents the rate of appearance of external glucose in the plasma glucose compartment due to food intake or exogenous glucose infusion intravenously. The model parameter, $p_{1}$ (min^−1^), and the ratio $p_{3}/p_{2}$ (l/(min×mU)) represent insulin‐independent glucose utilisation and insulin sensitivity, respectively. The parameter $p_{4}$ (min^−1^) stands for the insulin degradation rate and $p_{5}$ (min^−1^) is the rate of appearance of meal disturbance in the plasma glucose compartment [30].

### 2.2 Control objectives

The main intent of APS is to regulate the plasma glucose concentration within the safe range (70−180mg/dl) automatically via external insulin infusion through insulin pump. This is based on the glucose measurements provided by sensor by avoiding any events of severe hypoglycaemia (<50mg/dl) and prolonged hyperglycaemia (>180mg/dl). The design of the adaptive feedback control law u(t) should be such that the T1DM patient's BG $x_{1}(t)$ tracks the reference glucose signal ${\hat{x}}_{1}(t)$ generated from a reference system, in the presence of parametric uncertainty and meal disturbance. The main control objectives are stated as follows:

- The plasma glucose concentration should not decrease <50 mg/dl in order to avoid severe hypoglycaemic instances.
- The plasma glucose concentration $x_{1}$ should be brought <180 mg/dl within 150 min after a meal to avoid post‐prandial hyperglycaemia.
- The BG should be regulated within the safe range of 70–180 mg/dl in the presence of parametric uncertainty and external meal disturbance.

## 3 Design of the adaptive feedback control law

**Fig. 1:**
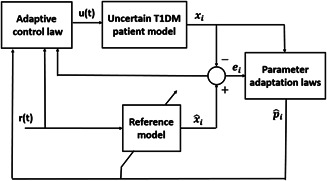
Adaptive closed‐loop control strategy

The conceptual framework of the proposed adaptive control technique is composed of (i) a reference model, (ii) parameter adaptation laws, and (iii) the adaptive control law as illustrated in Fig. 1.

Let us consider a reference system that is given by

(2) $$\begin{array}{rl} & {\dot{\hat{x}}}_{1}=-{\hat{p}}_{1}\left({\hat{x}}_{1}-G_{b}\right)-{\hat{x}}_{1}{\hat{x}}_{2}+{\hat{x}}_{4}\\ & {\dot{\hat{x}}}_{2}=-{\hat{p}}_{2}{\hat{x}}_{2}+{\hat{p}}_{3}\left({\hat{x}}_{3}-I_{b}\right)\\ & {\dot{\hat{x}}}_{3}=-{\hat{p}}_{4}\left({\hat{x}}_{3}-I_{b}\right)+r(t)\\ & {\dot{\hat{x}}}_{4}=-{\hat{p}}_{5}{\hat{x}}_{4} \end{array}$$

where ${\hat{p}}_{i},i=1,\ldots ,5$, are estimated parameters in the reference system, r(t) is the reference signal, and the reference states are ${\hat{x}}_{i},i=1,\ldots ,4$.

Subtracting (1) from (2), the error dynamics corresponding to the error signals $e_{i}={\hat{x}}_{i}-x_{i},\;i=1,\ldots ,4$, are obtained as follows:

(3) $$\begin{array}{rl} & {\dot{e}}_{1}=-{\tilde{p}}_{1}\left(x_{1}-G_{b}\right)-{\hat{p}}_{1}e_{1}-{\hat{x}}_{2}e_{1}+e_{4}-x_{1}e_{2}\\ & {\dot{e}}_{2}=-{\tilde{p}}_{2}x_{2}+{\tilde{p}}_{3}\left(x_{3}-I_{b}\right)-{\hat{p}}_{2}e_{2}+{\hat{p}}_{3}e_{3}\\ & {\dot{e}}_{3}=-{\tilde{p}}_{4}\left(x_{3}-I_{b}\right)-{\hat{p}}_{4}e_{3}+r-u\\ & {\dot{e}}_{4}=-{\tilde{p}}_{5}x_{4}-{\hat{p}}_{5}e_{4} \end{array}$$

where ${\tilde{p}}_{i}(t)={\hat{p}}_{i}(t)-p_{i},\;i=1,\ldots ,5$ are the parameter errors.

Now let us define the following adaptive update laws for estimating the parameters, ${\hat{p}}_{i},\;i=1,\ldots ,5$, of reference system (2)

(4) $$\begin{array}{rl} & {\dot{\hat{p}}}_{1}=c_{1}\gamma_{1}^{-1}e_{1}\left(x_{1}-G_{b}\right)\\ & {\dot{\hat{p}}}_{2}=c_{2}\gamma_{2}^{-1}e_{2}x_{2}\\ & {\dot{\hat{p}}}_{3}=-c_{2}\gamma_{3}^{-1}e_{2}\left(x_{3}-I_{b}\right)\\ & {\dot{\hat{p}}}_{4}=c_{3}\gamma_{4}^{-1}e_{3}\left(x_{3}-I_{b}\right)\\ & {\dot{\hat{p}}}_{5}=c_{4}\gamma_{5}^{-1}x_{4}e_{4}, \end{array}$$

and choose an adaptive control law u(t) given by

(5) $$u(t)=\frac{c_{2}}{c_{3}}{\hat{p}}_{3}(t)e_{2}(t)+r(t)$$

where $c_{i},\;i=1,\ldots ,5$, are certain constants which would be chosen so as to formulate constraints on certain estimated parameters.

*Note:* The specific formulations of the parameter adaptation laws (4) and the control law, u(t), in (5) are designed to ensure the stability of the proposed control technique by using Lyapunov's stability theorem.

The adaptive control scheme with control law u(t) in (5) along with the adaptive laws in (4), when applied to non‐linear error dynamics (3), ensures that all the closed‐loop signals of the system are bounded and the tracking errors asymptotically approach zero, that is, $\underset{t\to \infty }{\lim}e_{i}(t)=0,\;i=1,\ldots ,4$ [28, 29].

We choose a positive definite Lyapunov function candidate *V* given by

(6) $$V=\frac{1}{2}\sum_{i=1}^{4}c_{i}e_{i}^{2}+\frac{1}{2}\sum_{i=1}^{5}\gamma_{i}{\tilde{p}}_{i}^{2}$$

where $c_{i}>0,\;i=1,\ldots 4,\;\gamma_{i}>0,\;i=1,\ldots 5$.

After differentiating *V* in (6), we get

(7) $$\dot{V}=\frac{1}{2}\sum_{i=1}^{4}c_{i}{\dot{e}}_{i}^{2}+\frac{1}{2}\sum_{i=1}^{5}\gamma_{i}{\dot{\tilde{p}}}_{i}^{2}$$

Since ${\tilde{p}}_{i}(t)={\hat{p}}_{i}(t)-p_{i},\;i=1,\ldots ,5$, so by differentiating ${\tilde{p}}_{i}(t)$, we obtain

$${\dot{\tilde{p}}}_{i}(t)={\dot{\hat{p}}}_{i}(t)-{\dot{p}}_{i}={\dot{\hat{p}}}_{i}(t),\;i=1,\ldots ,5$$

where ${\dot{p}}_{i}$ vanishes as *p_i_* s are constant terms. Now, substituting the values of ${\dot{e}}_{i}$ from (3) and ${\dot{\tilde{p}}}_{i}(t)$ from (7), we have

(8) $$\begin{array}{rl} \dot{V} & =-c_{1}e_{1}{\tilde{p}}_{1}\left(x_{1}-G_{b}\right)-c_{1}{\hat{p}}_{1}e_{1}^{2}+c_{1}e_{1}e_{4}-c_{1}e_{1}x_{1}e_{2}\\ & \;-c_{2}{\hat{x}}_{2}e_{1}^{2}-c_{2}{\hat{p}}_{2}e_{2}^{2}-c_{2}{\tilde{p}}_{2}x_{2}e_{2}+c_{2}{\tilde{p}}_{3}\left(x_{3}-I_{b}\right)e_{2}\\ & \;+c_{2}{\hat{p}}_{3}e_{3}e_{2}-c_{3}e_{3}{\tilde{p}}_{4}\left(x_{3}-I_{b}\right)-c_{3}{\hat{p}}_{4}e_{3}^{2}-c_{3}e_{3}u\\ & \;-c_{4}{\hat{p}}_{5}e_{4}^{2}-c_{4}{\tilde{p}}_{5}x_{4}e_{4}+c_{3}re_{3}+\gamma_{1}{\tilde{p}}_{1}{\dot{\hat{p}}}_{1}+\gamma_{2}{\tilde{p}}_{2}{\dot{\hat{p}}}_{2}\\ & \;+\gamma_{3}{\tilde{p}}_{3}{\dot{\hat{p}}}_{3}+\gamma_{4}{\tilde{p}}_{4}{\dot{\hat{p}}}_{4}+\gamma_{5}{\tilde{p}}_{5}{\dot{\hat{p}}}_{5} \end{array}$$

The Lyapunov stability theorem requires that $\dot{V}$ should be negative definite function. Therefore, in the above expression for cancelling out the terms involving ${\tilde{p}}_{i},\;i=1,\ldots ,5$, we define ${\dot{\hat{p}}}_{i},\;i=1,\ldots ,5$ as in (4). Substituting ${\dot{\hat{p}}}_{i},\;i=1,\ldots ,5$ in (8), we get

(9) $$\begin{array}{rl} \dot{V} & =-c_{1}{\hat{p}}_{1}e_{1}^{2}+c_{1}e_{1}e_{4}-c_{4}{\hat{p}}_{5}e_{4}^{2}-c_{1}{\hat{x}}_{2}e_{1}^{2}-c_{1}e_{1}x_{1}e_{2}\\ & \;-c_{2}{\hat{p}}_{2}e_{2}^{2}+c_{2}{\hat{p}}_{3}e_{3}e_{2}-c_{3}{\hat{p}}_{4}e_{3}^{2}-uc_{3}e_{3}+rc_{3}e_{3} \end{array}$$

From (9), let us consider $V_{1}$ as

(10) $$\begin{array}{rl} V_{1} & =-c_{1}{\hat{p}}_{1}e_{1}^{2}+c_{1}e_{1}e_{4}-c_{4}{\hat{p}}_{5}e_{4}^{2}\\ & =-c_{1}{\hat{p}}_{1}{\left(e_{1}-\frac{e_{4}}{2{\hat{p}}_{1}}\right)}^{2}+c_{1}\frac{e_{4}^{2}}{4{\hat{p}}_{1}}-c_{4}{\hat{p}}_{5}e_{4}^{2} \end{array}$$

From (10), we obtain the following stability constraint in terms of $c_{1}$ and $c_{4}$

(11) $$\frac{c_{1}}{c_{4}}<4{\hat{p}}_{1}(t){\hat{p}}_{5}(t)$$

such that it renders $V_{1}\leq 0$ for all times. Again from (9), we consider $V_{2}$ as

(12) $$\begin{array}{rl} V_{2} & =-c_{1}{\hat{x}}_{2}e_{1}^{2}-c_{1}x_{1}e_{1}e_{2}-c_{2}{\hat{p}}_{2}e_{2}^{2}\\ & =-c_{2}{\hat{p}}_{2}{\left(e_{2}+\frac{c_{1}x_{1}e_{1}}{2c_{2}{\hat{p}}_{2}}\right)}^{2}+\frac{c_{1}^{2}x_{1}^{2}e_{1}^{2}}{4c_{2}{\hat{p}}_{2}}-c_{1}{\hat{x}}_{2}e_{1}^{2} \end{array}$$

For ensuring $V_{2}\leq 0$, another stability constraint in terms of $c_{1}$ and $c_{2}$ is obtained as

(13) $$\frac{c_{1}}{c_{2}}<\frac{4{\hat{p}}_{2}(t){\hat{x}}_{2}(t)}{x_{1}^{2}(t)}$$

It is clear that for guaranteeing negative definiteness of $V_{2}$, the continuous computation of the state $x_{1}(t)$, that is, the plasma glucose concentration can be easily obtained from the glucose sensors. Since the parameter estimates ${\hat{p}}_{i}(t),\;i=1,\ldots ,5$, and ${\hat{x}}_{2}(t)$ are positive values, substituting (10) and (12) in (9), we obtain

$$\dot{V}=V_{1}+V_{2}+c_{2}{\hat{p}}_{3}e_{3}e_{2}-c_{3}{\hat{p}}_{4}e_{3}^{2}-uc_{3}e_{3}+rc_{3}e_{3}$$

It is already proven that if the two stability constraints (11) and (13) are satisfied then $V_{1}$ and $V_{2}$ are guaranteed to be negative definite function. By neglecting the terms that are definitely negative and considering the other terms, the above equation can be written as

(14) $$\dot{V}\leq c_{2}{\hat{p}}_{3}e_{3}e_{2}-uc_{3}e_{3}+rc_{3}e_{3}$$

Now the choice of control law u(t) should be such that $\dot{V}<0$. Hence, if we substitute the adaptive control law given by (5) in (14), we can easily ensure

(15) $$\dot{V}\leq 0$$

Thus, the Lyapunov stability theorem guarantees $e_{i}(t),\;i=1,\ldots ,4$, and ${\hat{p}}_{i}(t),\;i=1,\ldots ,5$ to be uniformly bounded, and $e(t)\in L_{2}$ space. Finally, we conclude that $\underset{t\to \infty }{\lim}\;e_{i}(t)=0$. □

## 4 Numerical simulations

In this section, simulation studies are carried out to validate the proposed adaptive control algorithm for APS that is applied to system (1), to examine the effectiveness of the controller in regulating plasma glucose concentration in T1DM patients within a safe range in the presence of external meal disturbance and parametric uncertainties. The controller gains are provided in Table 1.

**Table 1:** Controller gains

Gains	Values	Gains	Values
*c*_1_	$8\times 10^{-4}$	*c*_2_	$5\times 10^{7}$
*c*_3_	$2\times 10^{-3}$	*c*_4_	$5\times 10^{4}$
$\gamma_{1}$	$5\times 10^{10}$	$\gamma_{2}$	$1\times 10^{8}$
$\gamma_{3}$	$1\times 10^{15}$	$\gamma_{4}$	$1\times 10^{5}$
$\gamma_{5}$	$2.7\times 10^{8}$	—	—

The parameters $c_{i},\;i=1,\ldots ,4$, are first fixed to ensure closed‐loop stability by satisfying the two stability constraints that are derived from the Lyapunov stability analysis. After fixing the $c_{i}$s, the parameters $\gamma_{i},\;i=1,\ldots ,5$, are then tuned heuristically for the finite‐time convergence of the estimated parameters to a stable value, thereby ensuring their boundedness. The reference signal is considered to be a function of the BG level and is given as

(16) $$r(t)=0.09x_{1}-6.5.$$

Three simulation scenarios have been proposed for corroboration of the adaptive control strategy as discussed subsequently.

### 4.1 Simulations with single meal disturbance under parametric uncertainty

#### 4.1.1 Objective

The main objective of the first simulation scenario is to assess the controller's ability to stabilise the states of the non‐linear model in a finite time under parametric uncertainty.

#### 4.1.2 Protocol

A single meal disturbance is provided at the start of the simulation and the initial plasma glucose concentration of T1DM patients are considered to be 250 mg/dl (hyperglycaemia) at *t* = 0 min. In order to indicate that there are no prior insulin dosages and high meal disturbance present, the initial conditions of the states $x_{2}=0\;\text{mi}n^{-1}$ (remote insulin), $x_{3}=7\;\text{mU}/l$ (basal insulin value), and $x_{4}=10$ (meal disturbance) are considered, respectively. In order to create a realistic scenario, the model parameters are considered uncertain and are randomly chosen from a specified range as provided in Table 2. The parameter $p_{1}$ is negligible in T1DM patients and hence, a very small value $p_{1}=1\times 10^{-7}$ is considered.

**Table 2:** Nominal and range of parameters for model (1)

Parameters	Values	Range
*p*_2_	0.015	[0.0105, 0.0195]
*p*_3_	$2\times 10^{-6}$	[$1.4\times 10^{-6}$, $2.6\times 10^{-6}$]
*p*_4_	0.2	[0.14, 0.26]
*p*_5_	0.05	[0.045, 0.055]

Important physiological factors like insulin sensitivity and insulin degradation rate in the blood plasma vary within a population of T1DM patients. A parametric uncertainty range of ±30% is considered here, which is sufficient for the investigation of the proposed controller in the presence of parametric uncertainty. It is also reported in [6], that the insulin sensitivity in T1DM patients can vary up to ±30%. Hence variation in the parameters $p_{2}$, $p_{3}$, and $p_{4}$ are considered to be ±30%, whereas the variation in $p_{5}$ is considered to be ±10% since it represents intravenous (directly into veins) glucose administration having less uncertainty than the others.

#### 4.1.3 Discussion

**Fig. 2:**
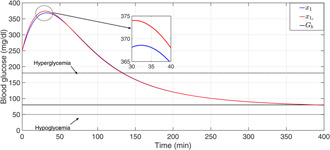
Closed‐loop BG profile for T1DM patient with nominal model parameters under the adaptive control law

**Fig. 3:**
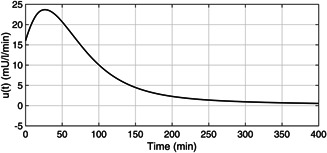
*Exogenous insulin infusion rate,* u(t), *as determined by the adaptive controller*

**Fig. 4:**
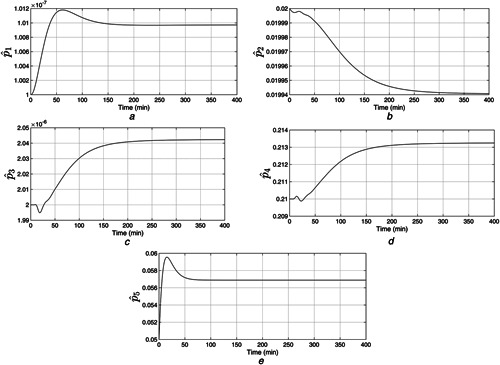
*Reference system parameters:* ${\hat{p}}_{i},\;i=1,\ldots ,5$

**Fig. 5:**
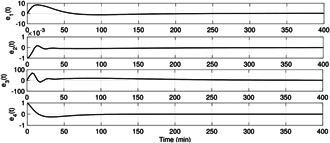
*Trajectories of error signals* $e_{i},\;i=1,\ldots ,4$, *showing the convergence to zero with time*

**Fig. 6:**
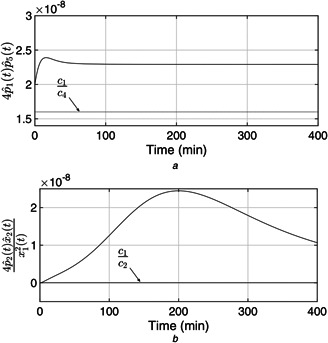
Stability constraints derive from Lyapunov stability analysis ***(a)*** $\left(c_{1}/c_{4})<4{\hat{p}}_{1}(t){\hat{p}}_{5}(t\right)$, ***(b)*** $\left(c_{1}/c_{2})<(4{\hat{p}}_{2}(t){\hat{x}}_{2}(t)/x_{1}^{2}(t)\right)$

The plasma glucose concentration is brought down from the hyperglycaemic level within 150 min and ultimately to the basal value $G_{b}=80\;\text{mg}/\text{dl}$ in the presence of high meal disturbance as depicted in Fig. 2. The attainment of the above control objective depends on the choice of r(t) which can be determined by exploiting the knowledge of the physician and clinical studies or conventional insulin therapy. In this work, the reference signal, r(t), in (16) exploits the information about $x_{1}$ such that $x_{1}$ is brought under 180 mg/dl within 150 min. Since the control law, u(t) as illustrated in Fig. 3, is a function of r(t), it follows the reference signal r(t) after some initial transients in order to achieve the above‐mentioned control objective. In addition to this, it is the parameter compensation via the parameter adaption laws that ensure the convergence of the outputs of both the uncertain and the reference systems to the basal value under parametric uncertainty as illustrated in Fig. 4. The convergence of error signals $e_{1}$, $e_{2}$, $e_{3}$, and $e_{4}$ to zero is shown in Fig. 5. Fig. 6 illustrates that during the whole simulation period, the stability constraints derived from the Lyapunov stability analysis are satisfied.

### 4.2 Simulations with multiple meal disturbances under parametric uncertainty

#### 4.2.1 Objective

A 3‐day (4320 min) long simulation scenario with three meal disturbances (breakfast, lunch, and dinner) at each day is considered here to assess the controller's performance in day‐to‐day glucose regulation in T1DM patients.

#### 4.2.2 Protocol

The simulation time 0 min corresponds to 12 AM of the first day of the simulation, and 1440 min to 12 AM of the next day. Meal disturbances of 5 mg/dl/min (breakfast), 8 mg/dl/min (lunch), and 8 mg/dl/min (dinner) are provided at 8 AM (480 min), 12 PM (720 min), and 8 PM (1200 min), respectively, and the same meal protocol is followed for the next 2 days. It is assumed that T1DM patients are in fasting state with no prior insulin infusions, which is reflected by the initial conditions $x_{1}=280\;\text{mg}/\text{dl}$, $x_{2}=0\;\text{mi}n^{-1}$, $x_{3}=7\;\text{mU}/l$, and $x_{4}=0\;\text{mg}/\text{dl}/\min$ (no initial meal disturbance). A total of 200 Monte Carlo simulations are carried out where the model parameters are chosen randomly.

#### 4.2.3 Discussion

T1DM patients have to reset the insulin pump frequently, as mentioned in [35], it is required to change the infusion set of the insulin pump in every 2–3 days. For this reason, a 3‐day scenario is considered here to investigate the robustness of the proposed algorithm with respect to the effect of multiple external meal disturbances as well as parametric uncertainty (±30%).

**Fig. 7:**
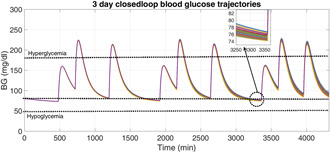
Closed‐loop BG trajectories of T1DM subjects with random model parameters with multiple meal disturbances for a simulation period of 3 days

**Fig. 8:**
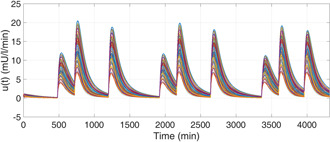
*Profile of exogenous insulin infusion rate,* u(t), *for 200 random T1DM subjects for a simulation period of 3 days*

It is evident from Fig. 7 that there are no episodes of severe hypoglycaemia or prolonged hyperglycaemia. The nature of the corresponding external insulin infusion rates determined by the adaptive controller is depicted in Fig. 8. Table 3 shows the percentage of total simulation time, where the BG profiles of different T1DM patients remain in the hypoglycaemic, safe range, and hyperglycaemic ranges. The closed‐loop BG trajectories are efficiently maintained within the safe range for 87.5% of the total time without any occurrences of hypoglycaemia. Post‐prandial hyperglycaemia is completely avoided, as the BG trajectories are brought <180 mg/dl after each of the meal disturbances within 120 min for each of the T1DM patients.

**Table 3:** Percentage of time of the closed‐loop BG trajectories

BG ranges	Percentage of time
hypoglycaemic range (<70 mg/dl)	0
safe range (70–180 mg/dl)	87.5
hyperglycaemic range (>180 mg/dl)	12.5

**Fig. 9:**
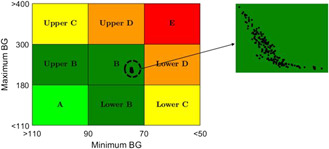
CVGA for 200 virtual T1DM subjects

Finally, in order to evaluate the efficacy of the proposed closed‐loop adaptive control scheme for the BG regulation in T1DM subjects in the presence of multiple meal disturbances and parametric uncertainty, control variability grid analysis (CVGA) is performed by carrying out 200 numerical simulations with random parameters selected from Table 1. As explained in [36], *Y*‐axis and *X*‐axis represent the maximum and minimum deviations of the BG during the whole simulation period. Here, 3‐day simulation for each of the random T1DM patients is represented by a black dot on CVGA plot as shown in Fig. 9. The zoomed image shows the distribution of the black dots. It can be observed that all the black dots are confined close to each other forming a cluster (encircled), and thus, a zoomed view of the cluster containing these black dots is provided for clarity. All the closed‐loop BG trajectories are confined to grid B (in green colour), indicating that all these closed‐loop trajectories remain within a safe range (with highest BG level <220 mg/dl and lowest BG level >75 mg/dl during the entire simulation) which is desirable. Thus, the proposed adaptive control algorithm is efficacious in preventing hypoglycaemic or hyperglycaemic events under parametric uncertainty and all closed‐loop results have a high degree of proximity as well as consistency.

In the current work, there exist two types of control challenges in the minimal model, (i) the external meal disturbances, and (ii) the parametric uncertainty. While the effect of the former is taken into account by considering meal disturbance as fourth state, $x_{4}$, the effect of the latter is expressed as parametric variability in the minimal model parameters representing important physiological factors like, insulin sensitivity ($p_{3}/p_{2}$), insulin degradation rate ($p_{4}$), and the time for maximum peak of the exogenous meal disturbance ($p_{5}$). The adaptive control approach employed here is able to successfully reject the effect of bounded meal disturbances and parametric uncertainty, so that the desired system performance can be achieved, thereby demonstrating the robustness of the proposed controller with respect to the above factors.

### 4.3 Simulations with unscheduled meal disturbance and noisy glucose measurements

#### 4.3.1 Objective

The sole objective of third simulation scenario is to test the controller's robustness with respect to unscheduled meal disturbance and the presence of noise in the measurements.

#### 4.3.2 Protocol

Initial conditions of the states are kept the same as in simulation scenario 1. Apart from the meal disturbance of 8 mg/dl/min at *t* = 0 min, an unscheduled meal (5 mg/dl/min) is introduced at *t* = 30 min. The glucose measurements coming from the sensor are assumed to be corrupted by a noise signal that is assumed to be normally distributed having zero mean and variance 20.

#### 4.3.3 Discussion

**Fig. 10:**
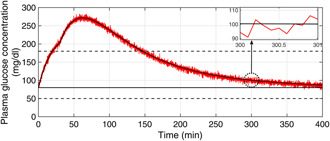
Closed‐loop BG trajectory of T1DM subject in the presence of sensor noise and unscheduled meal disturbance

**Fig. 11:**
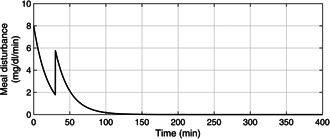
Profile of multiple meal disturbances where the first meal disturbance appears initially at t = 0 min and then an unscheduled meal disturbance is provided at t = 40 min

Despite the presence of unscheduled meal disturbances, the BG level is brought under 180 mg/dl within 150 min by the proposed adaptive controller in the presence of noisy glucose measurements as elucidated in Fig. 10. The red signal is the corrupted glucose measurement and the black trajectory is the actual BG level. The rate of appearance of the meal disturbance in the blood is depicted in Fig. 11.

## 5 Conclusion

A suitable yet simple adaptive state feedback control scheme is designed for a non‐linear intravenous T1DM patient model in the presence of parametric uncertainty. To account for the stability of the error during the design, an inherent parametric compensation technique is proposed within the adaptation law framework. The regulation of the glucose concentration and insulin injection profile under parametric uncertainty and meal disturbances are obtained for different patient scenarios through many random simulations. The results of several simulations are depicted via CVGA plot. The plot clearly reveals the reliability of the proposed control scheme in maintaining the profile of glucose concentration and insulin infusion similar to healthy subjects under parametric variability. Also it avoids occurrences of severe hypoglycaemic events. The advantage and flexibility in implementation of the proposed method practically will be more obvious when it is applied to a more complicated subcutaneous model and that will be treated as a future work by the authors.
